# Antiperistaltic effect and safety of l-menthol for esophagogastroduodenoscopy in the elderly with contraindication to hyoscine-*N*-butylbromide

**DOI:** 10.1038/s41598-022-14693-x

**Published:** 2022-06-21

**Authors:** Tsung-Chieh Yang, Ping-Hsien Chen, Ming-Chih Hou, Li-Ning Peng, Ming-Hsien Lin, Liang-Kung Chen, Yi-Hsiang Huang

**Affiliations:** 1 Division of Gastroenterology and Hepatology, Department of Medicine, Taipei Veterans General Hospital, #201, Section 2, Shipai Rd., Beitou District, Taipei, 11217 Taiwan; 2 School of Medicine, National Yang Ming Chiao Tung University, Taipei, Taiwan; 3 Endoscopy Center for Diagnosis and Treatment, Taipei Veterans General Hospital, Taipei, Taiwan; 4 Division of Gastroenterology and Hepatology, Department of Medicine, West Garden Hospital, Taipei, Taiwan; 5 Center for Geriatrics and Gerontology, Taipei Veterans General Hospital, Taipei, Taiwan; 6 Aging and Health Research Center, National Yang Ming Chiao Tung University, Taipei, Taiwan; 7 Taipei Municipal Gan-Dau Hospital, Taipei, Taiwan; 8 Institute of Clinical Medicine, National Yang Ming Chiao Tung University, Taipei, Taiwan

**Keywords:** Gastroenterology, Medical research

## Abstract

Hyoscine-*N*-butylbromide (HBB) is the most used antiperistaltic agent during esophagogastroduodenoscopy (EGD). However, almost half of the elderly have a contraindication to HBB. We aimed to evaluate l-menthol’s antiperistaltic effect and safety for EGD in the elderly with contraindication to HBB. This prospective, randomized, double-blind, placebo-controlled study screened 86 elderly patients (≥ 65 years old) scheduled to undergo EGD, and 52 of them with contraindication to HBB were enrolled. The participants were randomized to receive l-menthol (*n* = 26) or a placebo (*n* = 26), which was locally sprayed on the gastric antrum endoscopically. The proportion of patients with no or mild peristalsis after medication and at the end of EGD was significantly higher in the l-menthol group (76.9%) than in the placebo group (11.5%, *p* < 0.001). l-Menthol administration significantly reduced peristaltic grade, improved contraction parameters, and eased intragastric examination relative to the placebo (*p* < 0.001, respectively). Hemodynamic changes, adverse events, and discomfort levels of patients were similar between the two groups. l-Menthol is an effective and safe alternative antiperistaltic medication for EGD in elderly patients with contraindication to HBB. Further large, randomized trials are required to clarify whether l-menthol can lead to better detection yield in the elderly.

**Clinical trial registration:** The study was registered at ClinicalTrials.gov (NCT04593836).

## Introduction

Endoscopy plays an important role in the diagnosis and treatment of gastrointestinal diseases^1^. Effective suppression of gastrointestinal peristalsis during endoscopy is essential for optimal examination and lesion detection. In Taiwan, hyoscine-*N*-butylbromide (HBB) is the most used antispasmodic agent during endoscopic procedures. However, HBB can cause adverse drug reactions, such as dry mouth, palpitation, arrhythmia, blurred vision, urinary retention, allergic reactions and even deaths, limiting its application in the elderly^2–6^.

l-Menthol is the main component of peppermint oil, which is extracted from the natural plant (Mentha X piperita L) that grows in North America and Europe^7^. Animal studies indicated that menthol or peppermint oil exerted calcium channel blocking properties contributing to gastrointestinal smooth muscle relaxation^8,9^. Clinically, peppermint oil preparations have been widely used to relieve tension-type headache^10^, non-ulcer dyspepsia^11^, and irritable bowel syndrome symptoms^12–15^. Accumulating clinical trials have shown that direct endoscopic spraying peppermint oil or l-menthol on the gastrointestinal mucosa inhibited peristalsis and further improved the quality of colonoscopy^16,17^, barium enema^18,19^, endoscopic retrograde cholangiopancreatography^20^, and esophagogastroduodenoscopy (EGD)^21–23^.

With the aging of the global population, the proportion of elderly patients undergoing EGD is also increasing^24^. However, a large proportion of the elderly have multiple comorbidities, such as prostatic hyperplasia, heart disease and glaucoma, for which HBB is contraindicated^4^. It is crucial to find a safe alternative antiperistaltic medication for the elderly. Thus, we conducted a prospective, randomized, double-blind, placebo-controlled study that aimed to evaluate l-menthol’s antiperistaltic effect and safety for EGD in elderly patients who have a contraindication to HBB.

## Methods

### Patient selection and study design

This randomized controlled trial (RCT) enrolled participants in a tertiary medical center. All the elderly patients (≥ 65 years old) scheduled to undergo EGD were screened, and those with contraindication to HBB, such as prostatic hyperplasia, arrhythmia, ischemic heart disease and glaucoma, were consecutively enrolled. Patients were excluded if they had (1) allergy history to peppermint oil or l-menthol; (2) received radiotherapy or chemotherapy for cancer; (3) severe pyloric deformity or obstruction; (4) gastric or duodenal ulcers ≥ 2 cm in diameter; (5) gastric tumor; (6) upper gastrointestinal bleeding; (7) a history of gastric or duodenal surgery; or (8) severe comorbidities that were unsuitable for EGD. Eligible patients were randomized to receive a single dose of 160 mg l-menthol or placebo, which was sprayed on the gastric antrum during EGD, in a 1:1 ratio with variable block sizes. Randomization assignments were computer-generated and not announced until the trial was completed. To ensure blinding, treatment assignments were contained in sequentially numbered opaque sealed envelopes, which were opened by an independent research staff immediately after the patients’ eligibility was confirmed by endoscopy. The institutional review board of Taipei Veterans General Hospital approved this study (IRB number: 2011-07-016OB). The study was conducted following the ethical principles of the Declaration of Helsinki and Good Clinical Practice guidelines. All the participants signed the informed consent before enrollment. The study protocol was registered at ClinicalTrials.gov on 20/10/2020 (registration number: NCT04593836).

### Investigational drug preparation

According to the result of a phase-II study^22^, l-menthol suppresses peristalsis in a dose-dependent manner, and the dose–response reaches a plateau at 0.8% concentration. Therefore, we chose 0.8% l-menthol as the investigational drug in the experimental group. An 8-g volume of l-menthol crystals with a purity of at least 99% (Sigma-Aldrich Co, Ltd, Saint Louis, USA) and 8 g of Sorbitan monooleate (Spain 80) (Emperor Chemical Co, Ltd, Taipei, Taiwan), a common surface-active food additive, were mixed gently and dissolved in hot water. One liter of distilled deionized water was added to the dissolved l-menthol solution. The placebo solution was prepared with olive oil (Sigma-Aldrich Co, Ltd, Saint Louis, USA) in the same way as the l-menthol solution.

### Endoscopic procedure

The same endoscopist (P.-H.C.), who specialized in diagnostic endoscopy, performed all EGDs. The endoscopy room was pre-impregnated with the aroma of peppermint oil to ensure a double-blinded design. The gloves and masks worn by the endoscopist were also coated with peppermint oil. EGD was performed using a single-channel upper gastrointestinal endoscope (GIF-Q260 or GIF-H260, Olympus Medical Systems, Tokyo, Japan). The patients were not given systemic sedatives during the examination. The endoscopist checked the upper gastrointestinal tract first to ensure the eligibility of the patients. After that, the endoscope was kept in the gastric antrum (5 cm proximal to the pyloric ring). A 20-ml solution of 0.8% l-menthol (160 mg) or placebo was sprayed on the gastric antrum via the working channel of an endoscope according to the assignment. The residual fluid was pushed out by air.

**Figure 1:**
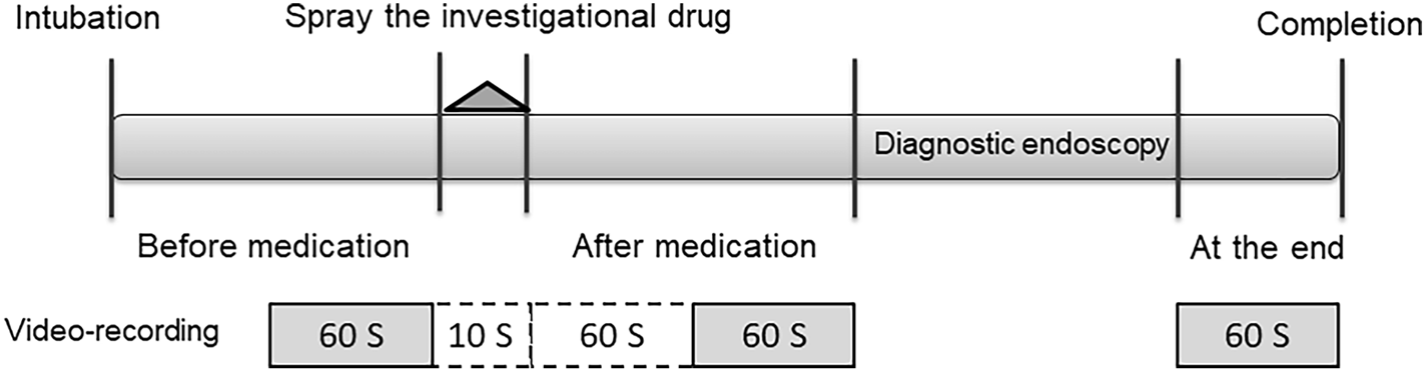
Schematic diagram of gastric peristalsis evaluation. Video images were recorded for three time periods: before medication (for 60 s), after medication (from 60 to 120 s after spraying the investigational drug), and at the end of esophagogastroduodenoscopy (for 60 s). The onset time of antiperistaltic effect was evaluated from 0 to 60 s after spraying the investigational drug.

**Figure 2:**
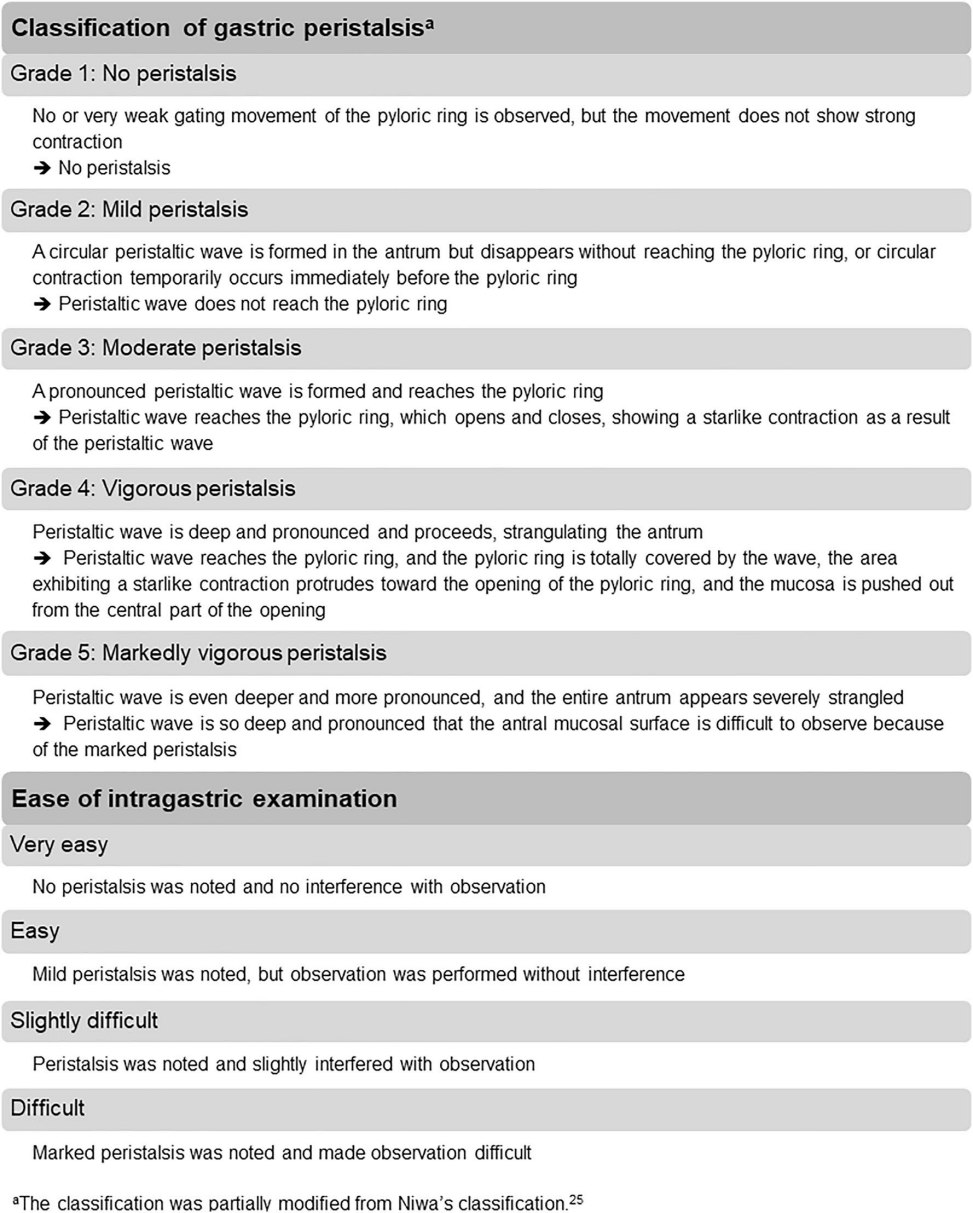
Evaluation of gastric peristalsis and ease of intragastric examination.

Endoscopic images of the pyloric ring and gastric antrum were videotaped for the following time periods: before medication (for 60 s), after medication (from 60 to 120 s after spraying the drug), and at the end of EGD (for 60 s) (Fig. 1). An independent research staff randomized the video images for each period. The randomized code for the video images was placed in an opaque sealed envelope until the trial was completed. Gastric peristalsis grade on video images was evaluated by another experienced endoscopist (T.-C.Y.) based on Hiki’s classification^23^, a version partially modified from Niwa’s classification^25^ (Fig. 2). The evaluator (T.-C.Y.) was blinded to the group assignment and the video record period. Standard gastric peristalsis grade on video images were evaluated by three endoscopists (T.-C.Y., P.-H.C. and M.-C.H.), and a consensus was reached before the study.

Before and 1 min after spraying the drug, the diameter of the pyloric ring in the maximally and minimally opened states were measured with an Olympus M2-4 K Measuring Device (Olympus Optical Co. Ltd, Tokyo, Japan). The antral contraction number per min was also recorded. The ease of intragastric examination was evaluated by the investigator (P.-H.C.) with a four-grade scale, according to whether gastric peristalsis after spraying the drug interfered with the observation (Fig. 2). After EGD, all patients reported on their discomfort level using the visual analog scale (VAS) and whether there were any adverse events (AEs).

### Outcomes

The proportion of patients with no (grade 1) or mild (grade 2) peristalsis after medication and at the end of EGD comprised the primary outcome. Peristaltic grade, contraction parameters, ease of intragastric observation, hemodynamic changes, AEs, and discomfort level of patients comprised the secondary outcomes.

### Definitions

Contraction ratio (%) was defined as (maximal pyloric ring diameter − minimal pyloric ring diameter) ÷ minimal pyloric ring diameter × 100. Opening ratio (%) of maximal/minimal pyloric ring was defined as (maximal/minimal pyloric ring diameter after medication − maximal/minimal pyloric ring diameter before medication) ÷ maximal/minimal pyloric ring diameter before medication × 100.

### Sample size calculation and statistical analysis

According to the result of a phase III study^23^, the proportion of patients with no or mild peristalsis at the end of EGD after medication was 77.8% in the l-menthol group and 35.7% in the placebo group. We set type I (α) error and type II (β) error to 0.05 and 0.2, respectively. The calculated sample size was 21 cases in each group by G*Power software, version 3.1.9.7 for Windows. It was estimated that 20% of patients would be lost to follow-up. Thus, the study would need to randomize 52 subjects.

Categorical variables were expressed as number (%), and analyzed by chi-square test using Yates’ correction, or Fisher exact test. Continuous variables for clinical characteristics and hemodynamic changes were expressed as mean ± standard deviation and analyzed by independent Student’s *t*-test. Continuous variables for peristaltic grade and contraction parameters were expressed as median [interquartile range (IQR)]. Differences of peristaltic grade and contraction parameters before and after medication within a group were analyzed by Wilcoxon signed-rank test, and differences between the two groups were analyzed by Mann–Whitney *U* test. *p* values < 0.05 were considered significant. All statistical analyses were performed using SPSS software, Windows version 23.0 (SPSS, Inc., Chicago, IL, USA).

## Results

### Study population

**Figure 3:**
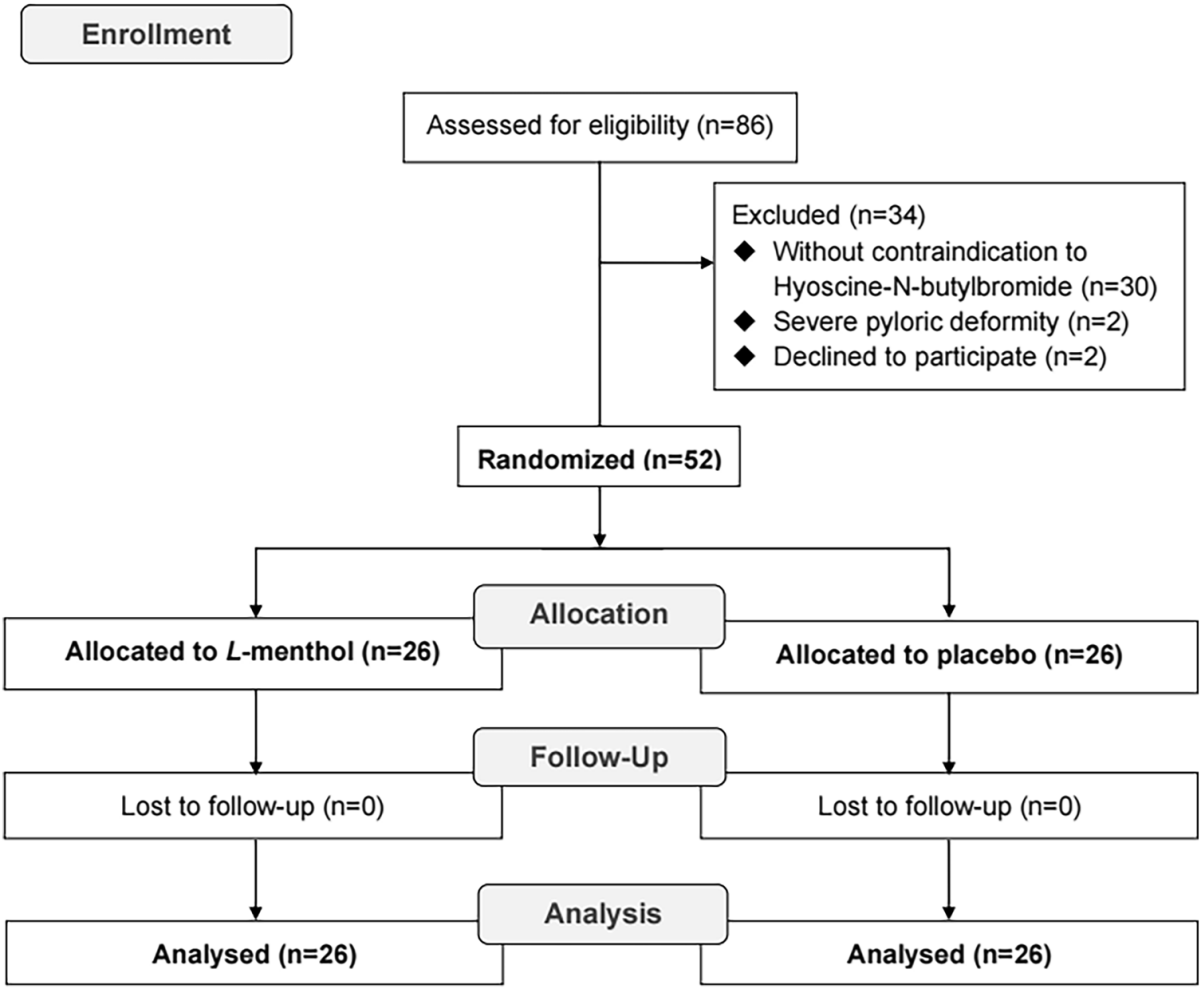
The Consolidated Standards of Reporting Trials (CONSORT) flow diagram of the study.

From March 2012 to March 2015, a total of 86 elderly patients scheduled to undergo EGD were screened (Fig. 3). Thirty-four patients were excluded (30 patients did not have contraindications to HBB, 2 patients had a severe pyloric deformity, and 2 patients declined to participate in this study). Fifty-two (60.5%) patients were consecutively enrolled and randomized into the l-menthol group (*n* = 26) and the placebo group (*n* = 26). No patient was lost to follow-up in both groups. All 52 patients were included in the final analysis.

Table 1 summarizes the clinical characteristics of the two groups. The overall mean patient age was 82.1 years, and male patients accounted for 67.3%. Prostatic hyperplasia (63.5%) was the most common cause of contraindications to HBB, followed by cardiac disease (36.5%) and glaucoma (19.2%). The most common endoscopic finding was esophagitis (63.5%), followed by gastritis (40.4%), gastric ulcer (19.2%) and duodenal ulcer (13.5%). The baseline characteristics did not differ between the two groups.

**Table 1:** Clinical characteristics of the two groups.

	l-menthol (*n* = 26)	Placebo (*n* = 26)	*p* value
Age, years	81.7 ± 7.8	82.6 ± 5.3	0.635
Male sex	18 (69.2)	17 (65.4)	1.000
**Causes of contraindications to HBB**			
Prostatic hyperplasia	17 (65.4)	16 (61.5)	1.000
Cardiac disease	8 (30.8)	11 (42.3)	0.565
Glaucoma	5 (19.2)	5 (19.2)	1.000
SBP, mmHg	149.2 ± 24.6	144.3 ± 23.6	0.465
DBP, mmHg	70.9 ± 15.5	68.5 ± 14.4	0.568
HR, bpm	70.1 ± 13.7	72.2 ± 11.3	0.553
Oxygen saturation, %	96.5 ± 2.3	97.5 ± 1.6	0.105
Examination time, min	9.7 ± 2.0	10.5 ± 2.0	0.163
**Endoscopic findings**			
Esophagitis	18 (69.2)	15 (57.7)	0.565
Gastritis	12 (46.2)	9 (34.6)	0.572
Gastric ulcer	4 (15.4)	6 (23.1)	0.725
Duodenal ulcer	3 (11.5)	4 (15.4)	1.000
Gastric polyp	2 (7.7)	3 (11.5)	1.000

Values are mean ± standard deviation or n (%).

*DBP* diastolic blood pressure, *HBB* hyoscine-*N*-butylbromide, *HR* heart rate, *SBP* systolic blood pressure.

### Primary outcome

**Figure 4:**
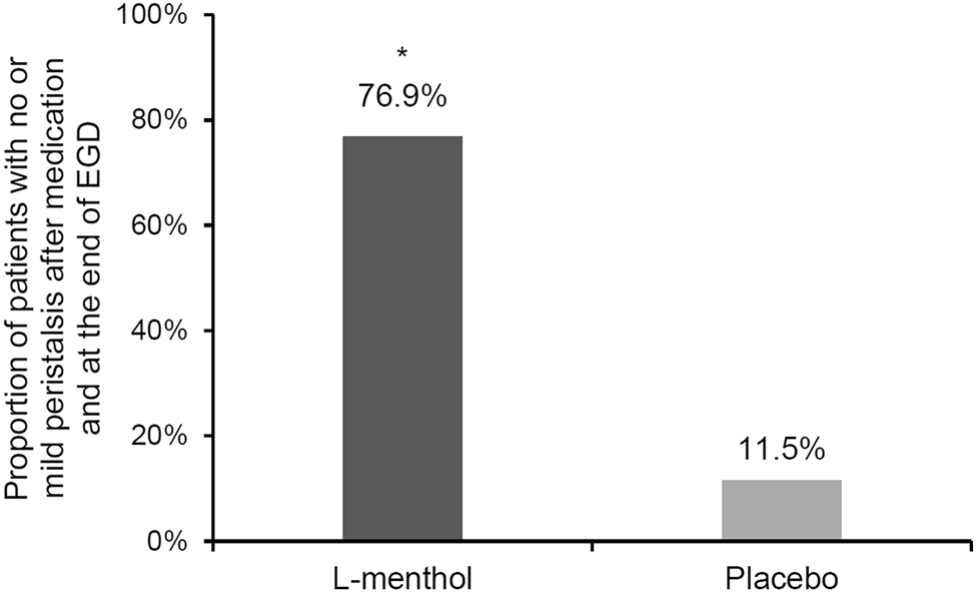
The proportion of patients with no (grade 1) or mild (grade 2) peristalsis after medication and at the end of EGD with l-menthol or placebo sprayed on the gastric mucosa. *p < 0.001. *EGD* esophagogastroduodenoscopy.

**Figure 5:**
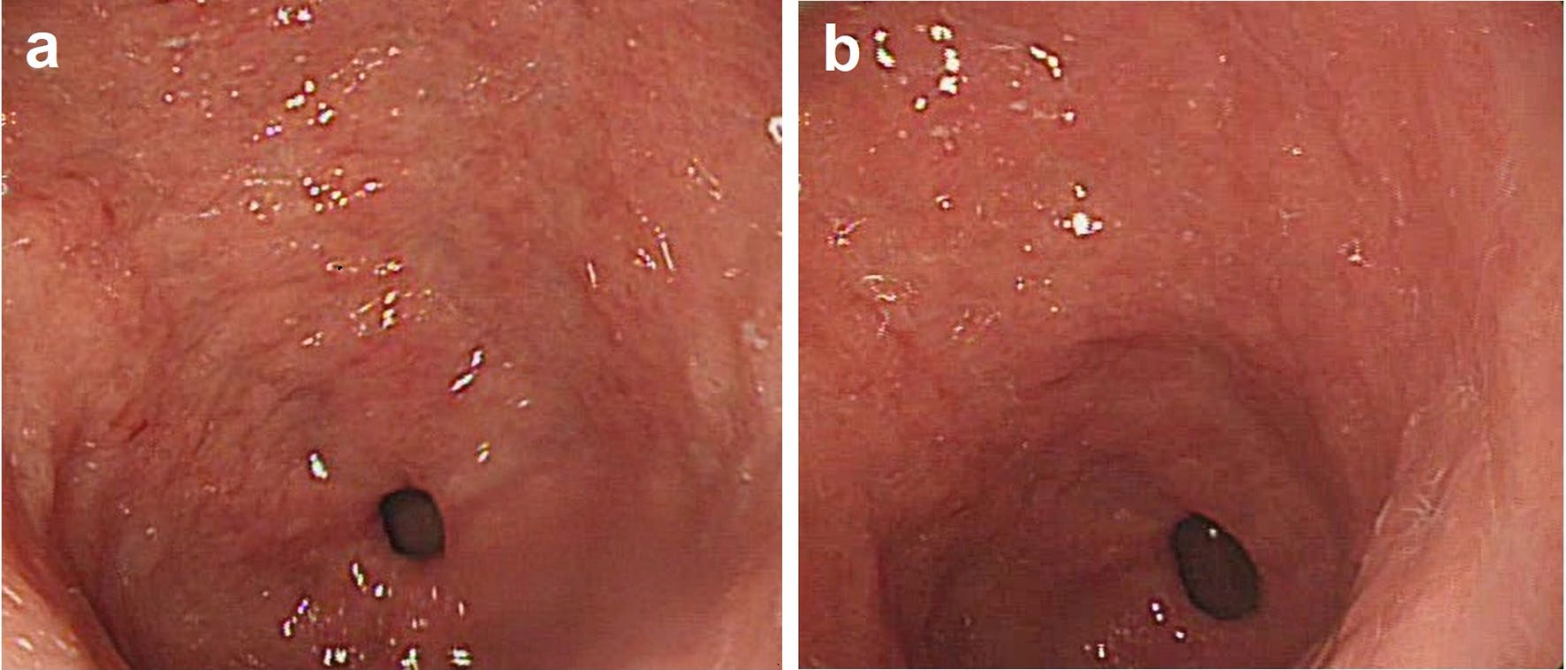
The representative endoscopic images before (**a**) and 1 min after spraying l-menthol (**b**).

The proportion of patients with no or mild peristalsis after medication and at the end of EGD was significantly higher in the l-menthol group (76.9%, 20/26 examinees) compared with the placebo group (11.5%, 3/26 examinees; *p* < 0.001; Fig. 4). The administration of l-menthol could quickly and obviously inhibit gastric peristalsis and relax the pylorus (Supplementary Video S1). The representative endoscopic images before and 1 min after spraying l-menthol were shown in Fig. 5.

### Secondary outcomes

#### Peristaltic grade

The number of patients categorized into each peristaltic grade in each period, as shown in Table 2. The peristaltic grade was converted into a numerical score for further analysis. Median (IQR) peristaltic score before medication was similar between the two groups [l-menthol: 4 (3–5), placebo: 4 (3–5); *p* = 0.601]. In the l-menthol group, median (IQR) peristaltic score was significantly lower after medication [1.5 (1–2.25); *p* < 0.001] and at the end of EGD [1 (1–2); *p* < 0.001] compared with that before medication [4 (3–5)]. In the placebo group, the score before medication did not differ from that after medication (*p* = 0.257) and at the end of EGD (*p* = 0.102). Compared with the placebo group, the l-menthol group had lower peristaltic scores both after medication and at the end of EGD (*p* < 0.001, respectively).

**Table 2:** Comparison of peristaltic grade in each period between the two groups.

	Peristaltic grade, *n* (%)					Median score (IQR)	*p* value (VS before medication)	*p* value (VS placebo)
	1	2	3	4	5			
**l-Menthol**								
Before medication	2 (7.7)	2 (7.7)	8 (30.8)	7 (26.9)	7 (26.9)	4 (3–5)		0.601
After medication	13 (50.0)	7 (26.9)	5 (19.2)	1 (3.8)	0 (0)	1.5 (1–2.25)	< 0.001	< 0.001
End of EGD	14 (53.8)	7 (26.9)	4 (15.4)	1 (3.8)	0 (0)	1 (1–2)	< 0.001	< 0.001
**Placebo**								
Before medication	2 (7.7)	1 (3.8)	6 (23.1)	10 (38.5)	7 (26.9)	4 (3–5)		
After medication	1 (3.8)	2 (7.7)	7 (26.9)	12 (46.2)	4 (15.4)	4 (3–4)	0.257	
End of EGD	1 (3.8)	2 (7.7)	8 (30.8)	11 (42.3)	4 (15.4)	4 (3–4)	0.102	

*EGD* esophagogastroduodenoscopy, *IQR* interquartile range, *VS* versus.

#### Contraction parameters

Table 3 presents the contraction parameters before and after medication in the two groups. Before spraying the drug, the contraction number [l-menthol: 3 (2–4), placebo: 4 (3–4)] and contraction ratio [l-menthol: 400 (100–1000), placebo: 900 (400–1900)] did not differ between the two groups (*p* = 0.118 and 0.124, respectively). After l-menthol administration, the contraction number [0 (0–2)] and contraction ratio [50 (0–100)] were significantly lower than those before medication (*p* < 0.001 and = 0.018, respectively). After placebo administration, however, there were no differences in the contraction number and contraction ratio compared with those before medication (*p* = 0.143 and 0.723, respectively). The contraction number and contraction ratio were both significantly lower after spraying l-menthol than placebo (*p* < 0.001, respectively). Moreover, opening ratio of minimal pyloric ring was also significantly higher in the l-menthol group [400 (0–1525)] than the placebo group [0 (0–75); *p* < 0.001].

**Table 3:** Comparison of contraction parameters, ease of intragastric examination, hemodynamic changes, visual analogue scale and adverse events between the two groups.

	l-menthol (*n* = 26)	Placebo (*n* = 26)	*p* value
**Contraction number per min**			
Before medication	3 (2–4)	4 (3–4)	0.118
After medication	0 (0–2)	3 (3–4)	< 0.001
*p* value	< 0.001	0.143	
**Contraction ratio, %**^**a**^			
Before medication	400 (100–1000)	900 (400–1900)	0.124
After medication	50 (0–100)	900 (250–1900)	< 0.001
*p* value	0.018	0.723	
Opening ratio of P-Max, %^b^	33.3 (4.2–100)	0 (0–72.9)	0.110
Opening ratio of P-Mini, %^c^	400 (0–1525)	0 (0–75)	< 0.001
**Ease of intragastric examination**			< 0.001
Very easy	15 (57.7)	2 (7.7)	
Easy	8 (30.8)	13 (50)	
Slightly difficult	3 (11.5)	11 (42.3)	
Difficult	0 (0)	0 (0)	
**Hemodynamic changes**^**d**^			
MD of SBP, mmHg	12.5 ± 23.8	10.3 ± 16.5	0.701
MD of DBP, mmHg	3.9 ± 13.3	2.4 ± 10.5	0.653
MD of HR, bpm	6.6 ± 8.3	4.9 ± 8.1	0.469
MD of oxygen saturation, %	0.0 ± 2.4	– 0.7 ± 1.5	0.234
Visual analog scale	2 (0–4)	3 (0–5.25)	0.385
**Adverse events**			
Overall	14 (53.8)	12 (46.2)	0.579
Dry mouth	6 (23.1)	4 (15.4)	0.726
Nausea	2 (7.7)	6 (23.1)	0.248
Dizziness	5 (19.2)	4 (15.4)	1.000
Palpitation	4 (15.4)	3 (11.5)	1.000
Urinary retention	1 (3.8)	2 (7.7)	1.000
Abdominal distention	5 (19.2)	8 (30.8)	0.523
Blurred vision	2 (7.7)	2 (7.7)	1.000
Heartburn	2 (7.7)	0 (0)	0.490
Headache	2 (7.7)	1 (3.8)	1.000

Values are median (interquartile range), mean ± standard deviation, or n (%).

*DBP* diastolic blood pressure, *HR* heart rate, *MD* mean difference, *SBP* systolic blood pressure.

^a^Contraction ratio, % = (maximal pyloric ring diameter − minimal pyloric ring diameter) ÷ minimal pyloric ring diameter × 100.

^b^Opening ratio of P-Max, % = (maximal pyloric ring diameter after medication − maximal pyloric ring diameter before medication) ÷ maximal pyloric ring diameter before medication × 100.

^c^Opening ratio of P-Mini, % = (minimal pyloric ring diameter after medication − minimal pyloric ring diameter before medication) ÷ minimal pyloric ring diameter before medication × 100.

^d^Mean difference = the mean of (max SBP or DBP or HR or oxygen saturation after medication − baseline SBP or DBP or HR or oxygen saturation before medication).

#### Ease of intragastric examination, hemodynamic changes, VAS and AEs

Comparison of ease of intragastric examination, hemodynamic changes, VAS and AEs between the two groups is shown in Table 3. The investigator evaluated the ease of intragastric examination to be very easy or easy in 88.5% (23/26 examinees) of the patients in the l-menthol group compared with 57.7% (15/26 examinees) in the placebo group (*p* < 0.001). There were no significant differences in the mean difference of systolic blood pressure, diastolic blood pressure, heart rate, and oxygen saturation between the two groups (*p* = 0.701, 0.653, 0.469, and 0.234, respectively). The discomfort level of patients assessed by VAS was similar between the two groups [l-menthol: 2 (0–4), placebo: 3 (0–5.25); *p* = 0.385]. The overall incidence of AEs did not differ between the two groups (l-menthol: 53.8%, 14/26 examinees; placebo: 46.2%, 12/26 examinees; *p* = 0.579). The most common AE was dry mouth (23.1%) in the l-menthol group and abdominal distention (30.8%) in the placebo group. All the AEs were mild and resolved on the next day of the examination. No serious complication or death was reported. There was no significant difference in any specific AE between the two groups.

## Discussion

The present study demonstrated that l-menthol sprayed on the gastric mucosa in the elderly with contraindication to HBB significantly inhibited gastric peristalsis, improved contraction parameters and eased intragastric examination relative to the placebo. The degree of hemodynamic changes, discomfort level of patients, and incidence of AEs were similar between the two groups. To our knowledge, this is the first RCT to prove the antiperistaltic effect and safety of l-menthol for EGD in the geriatric population.

EGD in geriatric patients is increasing as a larger proportion of the population is reaching an advanced age^24^. The mean patient age in the present study was up to 82.1 years, and no serious AEs or death relevant to EGD was reported, demonstrating the safety of EGD in the elderly. Elderly patients are also known to be at an increased risk of developing peptic ulcer disease and gastric cancer^26,27^. In this study, 32.7% of elderly patients were found to have gastric or duodenal ulcers, supporting the necessity of EGD in these examinees. Traditionally, HBB is commonly used as an antispasmodic agent during EGD. However, 60.5% of elderly patients screened in this study had contraindications to HBB. There is a need to identify a suitable alternative antispasmodic drug for the elderly with contraindication to HBB.

l-Menthol has been shown to effectively suppress gastric peristalsis with few AEs while intraluminally administered during EGD in the general population^22,23,28–30^. Although its application in elderly patients was mentioned in some studies, no well-designed RCT was conducted until now. A non-randomized trial showed that the antispasmodic effect of peppermint oil was similar to HBB in elderly patients, but inferior to HBB in non-elderly patients^31^. However, there was bias in this study because higher percentages of males and elderly people were noted in the peppermint oil group than the HBB group, and the endoscopists were aware of the drugs being administered. To overcome the inherent limitations of a non-randomized study, we designed this RCT to explore the antiperistaltic effect of l-menthol in the elderly.

The present study showed that the proportion of patients with no or mild peristalsis (sufficient suppression of gastric peristalsis) after medication and at the end of EGD was significantly higher in the l-menthol group than in the placebo group. In addition, the peristaltic score after spraying l-menthol was significantly lower than that after spraying placebo. These findings confirmed the antiperistaltic effect of l-menthol in the geriatric population. Furthermore, l-menthol had a fast antispasmodic effect (mean onset time: 20.2 s; data not shown) and persisted to the completion of the exam (80.7% of the examinees in the l-menthol group continued to have minimal peristalsis at the end of EGD). The evaluation of peristaltic grade, however, might be criticized as a subjective assessment method. Therefore, we also examined the contraction parameters, a more objective method, as a secondary outcome. The results showed that l-menthol administration significantly decreased the contraction number and contraction ratio, and increased opening ratio of minimal pyloric ring relative to the placebo, which objectively demonstrated the antispasmodic effect of l-menthol.

From the view of the endoscopist, the intragastric examination was significantly easier after administration of l-menthol than placebo. In the l-menthol group, the rate of very easy or easy examination (88.5%, 23/26 examinees) was comparable to the rate of minimal peristalsis at the end of EGD (80.7%, 21/26 examinees), suggesting that minimal peristalsis was acceptable for the endoscopist and did not interfere with observation in a clinical setting. From the view of the examinees, the discomfort level and hemodynamic changes were similar in the two groups. Half of the elderly had AEs after EGD, with the incidence rate higher than the results of previous studies enrolling the general population^22,23,28,29^. This is reasonable because the risk of complications of EGD was increased for elderly patients due to their underlying disease^31^. Importantly, all the AEs were mild and similar in the two groups, suggesting those were related to EGD itself rather than the drug effects.

There are some advantages of l-menthol as an antispasmodic agent in the elderly. First, l-menthol was extracted from the natural plant and was associated with a low risk of adverse drug reactions. Therefore, it was safer than conventional antispasmodic agents, especially in the elderly. Second, l-menthol inhibited gastric peristalsis with a rapid onset time and sustained for at least 10 min^21,32^. Third, the l-menthol preparation could be sprayed via the working channel of the endoscope easily and non-invasively. Furthermore, the l-menthol solution could be administered repeatedly during prolonged endoscopic procedures^33^. Finally, the pleasant aroma of l-menthol might have anti-anxiety and relaxing effects on the examinees.

The present study has several strengths. First, this was the first RCT demonstrating the antiperistaltic effect and safety of l-menthol for EGD in the geriatric population. Second, the clinical characteristics and baseline peristaltic grade were comparable in both study groups, thus eliminating selection bias. Third, we concurrently assessed peristaltic grade and contraction parameters as a subjective and an objective evaluation method, respectively, that made our findings more solid than previous studies. We also acknowledge some limitations in this study. First, this is a single-center RCT with a relatively small sample size. However, we had enrolled enough participants to achieve the calculated sample size, and definitely found a positive finding on the primary outcome. Second, the l-menthol preparation has not yet been commercialized in Taiwan, and the problem of unstable formulations during the catalyx process remains to be resolved. Furthermore, it may be questioned that whether the white, oily nature of l-menthol solution interferes with visibility during EGD. In our experience, l-menthol would be diluted by gastric juice or flowed to other locations within 1 min of being sprayed on the gastric antrum, so it had minimal interference on the endoscopic observation (Fig. 5). In fact, several previous studies even showed that spraying l-menthol onto lesions may facilitate the endoscopic clarification of pathological gastric lesions or early gastric cancer^34,35^. The impact of l-menthol on the defection of lesions during endoscopy needs to be clarified in further large, randomized trials.

In conclusion, the present study demonstrates that l-menthol is an effective and safe alternative antiperistaltic medication for EGD in elderly patients with contraindication to HBB. Further large, randomized trials are required to clarify whether l-menthol can lead to better detection yield in the elderly.

## Supplementary Information

Supplementary Information 1.

Supplementary Video 1.

## Data Availability

The datasets generated during and analyzed during the current study are available from the corresponding author on reasonable request.

## Abbreviations

AE: Adverse event
DBP: Diastolic blood pressure
EGD: Esophagogastroduodenoscopy
HBB: Hyoscine-*N*-butylbromide
HR: Heart rate
IQR: Interquartile range
MD: Mean difference
RCT: Randomized controlled trial
SBP: Systolic blood pressure
VAS: Visual analog scale
VS: Versus

## References

[1] Leung WK (2008). Screening for gastric cancer in Asia: Current evidence and practice. Lancet Oncol..

[2] Katoh K (2003). Comparison of gastric peristalsis inhibition by scopolamine butylbromide and glucagon: Evaluation by electrogastrography and analysis of heart rate variability. J. Gastroenterol..

[3] González-Mendiola R (2004). Acute urticaria induced by hyoscine butylbromide. Allergy.

[4] Umegaki E (2010). Risk management for gastrointestinal endoscopy in elderly patients: Questionnaire for patients undergoing gastrointestinal endoscopy. J. Clin. Biochem. Nutr..

[5] Treweeke P, Barrett NK (1987). Allergic reaction to Buscopan. Br. J. Radiol..

[6] Ikegaya H, Saka K, Sakurada K, Nakamura M, Yoshida K (2006). A case of sudden death after intramuscular injection of butylscopolamine bromide. Leg. Med. (Tokyo).

[7] Kligler B, Chaudhary S (2007). Peppermint oil. Am. Fam. Physician.

[8] Hawthorn M (1988). The actions of peppermint oil and menthol on calcium channel dependent processes in intestinal, neuronal and cardiac preparations. Aliment Pharmacol. Ther..

[9] Hills JM, Aaronson PI (1991). The mechanism of action of peppermint oil on gastrointestinal smooth muscle. An analysis using patch clamp electrophysiology and isolated tissue pharmacology in rabbit and guinea pig. Gastroenterology.

[10] Göbel H, Fresenius J, Heinze A, Dworschak M, Soyka D (1996). Effectiveness of Oleum menthae piperitae and paracetamol in therapy of headache of the tension type. Nervenarzt.

[11] May B, Kuntz HD, Kieser M, Köhler S (1996). Efficacy of a fixed peppermint oil/caraway oil combination in non-ulcer dyspepsia. Arzneimittelforschung.

[12] Cappello G, Spezzaferro M, Grossi L, Manzoli L, Marzio L (2007). Peppermint oil (Mintoil) in the treatment of irritable bowel syndrome: A prospective double blind placebo-controlled randomized trial. Dig. Liver Dis..

[13] Merat S (2010). The effect of enteric-coated, delayed-release peppermint oil on irritable bowel syndrome. Dig. Dis. Sci..

[14] Pittler MH, Ernst E (1998). Peppermint oil for irritable bowel syndrome: A critical review and metaanalysis. Am. J. Gastroenterol..

[15] Liu JH, Chen GH, Yeh HZ, Huang CK, Poon SK (1997). Enteric-coated peppermint-oil capsules in the treatment of irritable bowel syndrome: A prospective, randomized trial. J. Gastroenterol..

[16] Asao T (2001). An easy method for the intraluminal administration of peppermint oil before colonoscopy and its effectiveness in reducing colonic spasm. Gastrointest. Endosc..

[17] Leicester RJ, Hunt RH (1982). Peppermint oil to reduce colonic spasm during endoscopy. Lancet.

[18] Asao T (2003). Spasmolytic effect of peppermint oil in barium during double-contrast barium enema compared with Buscopan. Clin. Radiol..

[19] Sparks MJ, O'Sullivan P, Herrington AA, Morcos SK (1995). Does peppermint oil relieve spasm during barium enema?. Br. J. Radiol..

[20] Yamamoto N (2006). Efficacy of peppermint oil as an antispasmodic during endoscopic retrograde cholangiopancreatography. J. Gastroenterol. Hepatol..

[21] Hiki N (2003). Peppermint oil reduces gastric spasm during upper endoscopy: A randomized, double-blind, double-dummy controlled trial. Gastrointest. Endosc..

[22] Hiki N (2012). Multicenter phase II randomized study evaluating dose-response of antiperistaltic effect of L-menthol sprayed onto the gastric mucosa for upper gastrointestinal endoscopy. Dig. Endosc..

[23] Hiki N (2011). Antiperistaltic effect and safety of L-menthol sprayed on the gastric mucosa for upper GI endoscopy: A phase III, multicenter, randomized, double-blind, placebo-controlled study. Gastrointest. Endosc..

[24] Chandrasekhara V (2013). Modifications in endoscopic practice for the elderly. Gastrointest. Endosc..

[25] Niwa H, Nakamura T, Fujino M (1975). Endoscopic observation on gastric peristalsis and pyloric movement [in Japanese with English abstract]. Gastroenterol. Endosc..

[26] Franceschi M, Di Mario F, Leandro G, Maggi S, Pilotto A (2009). Acid-related disorders in the elderly. Best Pract. Res. Clin. Gastroenterol..

[27] Pilotto A, Franceschi M, Maggi S, Addante F, Sancarlo D (2010). Optimal management of peptic ulcer disease in the elderly. Drugs Aging.

[28] Hiki N (2011). An open-label, single-arm study assessing the efficacy and safety of L: -menthol sprayed onto the gastric mucosa during upper gastrointestinal endoscopy. J. Gastroenterol..

[29] Meng F (2021). Antiperistaltic effect and safety of l-menthol oral solution on gastric mucosa for upper gastrointestinal endoscopy in Chinese patients: Phase III, multicenter, randomized, double-blind, placebo-controlled study. Dig. Endosc..

[30] You Q (2020). L-menthol for gastrointestinal endoscopy: A systematic review and meta-analysis. Clin. Transl. Gastroenterol..

[31] Imagawa A (2012). Peppermint oil solution is useful as an antispasmodic drug for esophagogastroduodenoscopy, especially for elderly patients. Dig. Dis. Sci..

[32] Hiki N (2011). A phase I study evaluating tolerability, pharmacokinetics, and preliminary efficacy of L-menthol in upper gastrointestinal endoscopy. Clin. Pharmacol. Ther..

[33] Fujishiro M (2014). Efficacy of spraying l-menthol solution during endoscopic treatment of early gastric cancer: A phase III, multicenter, randomized, double-blind, placebo-controlled study. J. Gastroenterol..

[34] Mori A (2014). l-Menthol sprayed on gastric mucosa causes edematous change. Endosc. Int. Open.

[35] Kikuchi H (2021). Effectiveness of L-menthol spray application on lesions for the endoscopic clarification of early gastric cancer: Evaluation by the color difference. Digestion.

