# Supplementary material for: Antiperistaltic effect and safety of l-menthol for esophagogastroduodenoscopy in the elderly with contraindication to hyoscine-N-butylbromide
Source: Sci Rep. 2022 Jun 21;12:10418. doi: 10.1038/s41598-022-14693-x (PMC9213511; doi:10.1038/s41598-022-14693-x)
Supplement: Supplementary file 1 — Supplementary Information 1. [file 41598_2022_14693_MOESM1_ESM.docx]

**Supplementary video legends**

**Supplementary Video S1.** The administration of L-menthol could quickly and obviously inhibit gastric peristalsis and relax the pylorus.
